# CpG promoter hypo-methylation and up-regulation of microRNA-190b in hormone receptor-positive breast cancer

**DOI:** 10.18632/oncotarget.27083

**Published:** 2019-07-23

**Authors:** Elisabet Frick, Thorkell Gudjonsson, Jorunn Eyfjord, Jon Jonasson, Laufey Tryggvadóttir, Olafur Stefansson, Stefan Sigurdsson

**Affiliations:** ^1^Cancer Research Laboratory, Biomedical Center, Reykjavik, Iceland; ^2^Faculty of Medicine, University of Iceland, Reykjavik, Iceland; ^3^Department of Biochemistry and Molecular Biology, Biomedical Center, Reykjavik, Iceland; ^4^Department of Pathology, Landspitali University Hospital, Reykjavik, Iceland; ^5^Icelandic Cancer Registry, Reykjavik, Iceland; ^6^Current affiliation: deCODE genetics/Amgen Inc., Reykjavik, Iceland

**Keywords:** breast cancer, microRNA-190b, DNA methylation, prognosis, estrogen receptor

## Abstract

Estrogen receptor-positive breast cancer is subdivided into subtypes LuminalA and LuminalB, based on different expression patterns. MicroRNA-190b has been reported to be up-regulated in estrogen receptor-positive breast cancers. In this study we aimed to investigate the role of CpG promoter methylation in regulating miR-190b expression and its impact on clinical presentation and prognosis. DNA methylation analysis for the promotor of microRNA-190b was performed by pyrosequencing 549 primary breast tumors, of which 62 were carriers of the *BRCA2*^*999del5*^ founder mutation, 71 proximal normal breast samples and 16 breast derived cell lines. MicroRNA-190b expression was analysed in 67 primary breast tumors, 14 paired normal breast samples and 16 breast derived cell lines. Tissue microarrays (TMAs) were available for ER (*n* = 436), PR (*n =* 436), HER-2 (*N* = 258) and Ki67 (*n =* 248). MiR-190b had reduced promoter methylation in estrogen receptor-positive breast cancers (*P =* 1.02e–12, Median values: ER+ 24.3, ER– 38.26) and miR-190b’s expression was up-regulated in a correlative manner (*P =* 1.83e–06, Spearman’s rho –0.62). Through breast cancer specific survival analysis, we demonstrated that LuminalA patients exhibiting miR-190b hypo-methylation had better survival than other patients (*P* = 0.034, HR = 0.29, 95% CI 0.09-0.91). We, furthermore, demonstrated that miR-190b hypo-methylation occurs less frequently in ER+ tumors from *BRCA2*^*999del5*^ mutation carriers than in non-mutated individuals (*P =* 0.038, *Χ*^2^ = 4.32, *n =* 335). Our results suggest that upregulation of miR-190b may occur through loss of promoter DNA methylation during the development of estrogen-receptor (ER) positive breast cancers, and that miR-190b hypo-methylation leads to increased breast cancer specific survival within the LuminalA- subtype but not LuminalB.

## INTRODUCTION

Breast cancer is a complex, heterogeneous disease with at least five subtypes defined on the basis of genome-wide expression patterns [1–3]. These subtypes are thought to emerge through distinct tumor evolutionary paths and due to their diverse clinical outcome, patient prognosis is highly dependent on tumor subtype [4].

Estrogen receptor-positive (ER+) breast cancer is the most common form of breast cancer diagnosed representing approximately 70% of total incidences, and is rapidly becoming the most commonly diagnosed malignancy worldwide [5–7]. ER+ breast cancers, which are classified as luminal subtypes LuminalA (LumA) and LuminalB (LumB), are most commonly treated using agents inhibiting the estrogen receptor or hormone levels [8, 9]. These cancers have fairly good prognosis, though a subset of patients respond poorly to treatment. This is particularly relevant for LumB type breast cancers, which are diagnosed in younger patients, have higher tumor-proliferation rates and have worse prognosis compared to LumA patients [5, 10, 11]. Although LumA and LumB breast cancers are in general classified by defined markers, their full biological distinction regarding treatment remains poorly understood. Recent studies have shown that LumA and LumB breast cancers have several seperate features, and that the growth of these tumors is driven by different oncogenic mechanisms [10]. To distinguish between the two cancer groups is thus important for clinical practice [5]. It is necessary to fully study the Luminal subtypes for better understanding of the oncogenic mechanisms driving these cancers and improving patient outcomes.

The Icelandic *BRCA*^*999del5*^ founder mutation (c.771_775del5) has a prevalence of approximately 6–7% in Icelandic female breast cancer patients. It is a pathogenic mutation, associated with an increased risk of breast-, ovarian- and other cancers. Patients with this mutation have been reported to have poorer prognosis than non-carriers, although age of onset and disease severity differs between individuals [12–14].

MicroRNAs (miRNA) are small non-coding RNA molecules with an important role in post-transcriptional gene silencing via sequence-specific interaction with the 3’UTR of mRNA. MiRNAs are important for fine tuning gene translation and their expression is often tissue specific [15], they can influence multiple genes simultaneously and have widespread phenotypic impact [16, 17].

Abnormal miRNA expression has been observed in cancer and multiple studies have shown that miRNA expression abnormalities are causatively linked to carcinogensis [18–20]. MiR-190b has been reported to be up-regulated in ER+ breast cancers [21]. However, little is known about the mechanism underlying miR-190b up-regulation or its impact on clinical presentation and prognosis.

In this study, we show that miR-190b promoter methylation loss in tumors is strongly associated with miR-190b over-expression and that breast cancer specific survival is better in individuals with hypo-methylated breast tumors of subtype LumA.

## RESULTS

### MiR-190b expression in breast derived cell lines

**Figure 1:**
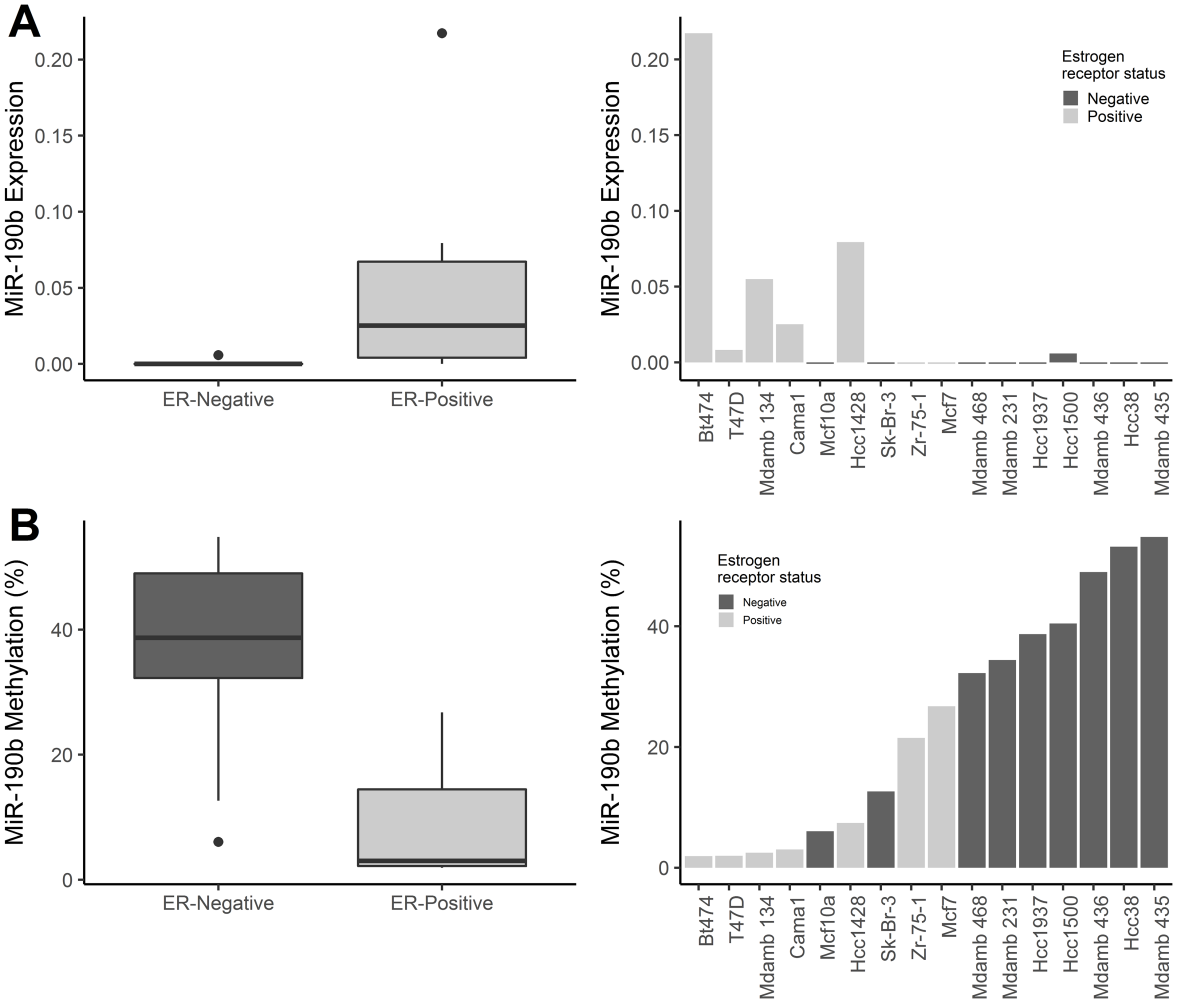
MiR-190b expression and methylation status in breast derived cell lines. (**A**, left panel) MiR-190b expression is higher in ER+ breast cancer cell lines (*n* = 7) compared to ER– breast cancer cell lines (*n* = 9) (Wilcoxon rank sum test *P* = 0.011). Right panel- MiR-190b expression in breast cancer cell lines. (**B**, left panel) MiR-190b methylation is lower in ER+ (*n* = 7) compared to ER– cell lines (*n* = 9) (Wilcoxon rank sum test, *P* = 0.003). (Right panel) MiR-190b methylation in breast cancer cell lines.

MiR-190b has been shown to be up-regulated in ER+ breast cancers, however, the mechanisms behind this up-regulation is unknown. Therefore, we analysed miR-190b‘s expression and methylation status in breast derived cell lines (*n* = 16). ER+ cell lines displayed overall higher miR-190b expression compared to ER– cell lines (Wilcoxon rank sum test *P* = 0.011, Median values ER+ 0.025, ER– 0) (Figure 1A). We pyrosequenced the first CpG upstream from miR-190b‘s genomic sequence (Supplementary Figure 1) in the same set of 16 cell lines and found that ER+ cell lines were significantly less methylated comparing to ER– cell lines. (Wilcoxon rank sum test, *P* = 0.003, Median values ER+ 3.02, ER– 38.71) (Figure 1B). MiR-190b methylation and expression were also significantly correlated (Spearman’s rho = –0.68, *P* = 0.004) (Supplementary Figure 2). Following our findings, we proceeded to investigate miR-190b‘s expression and methylation status within patient derived breast tumor samples.

### MiR-190b expression in ER+ and ER– breast cancer tumors

**Figure 2:**
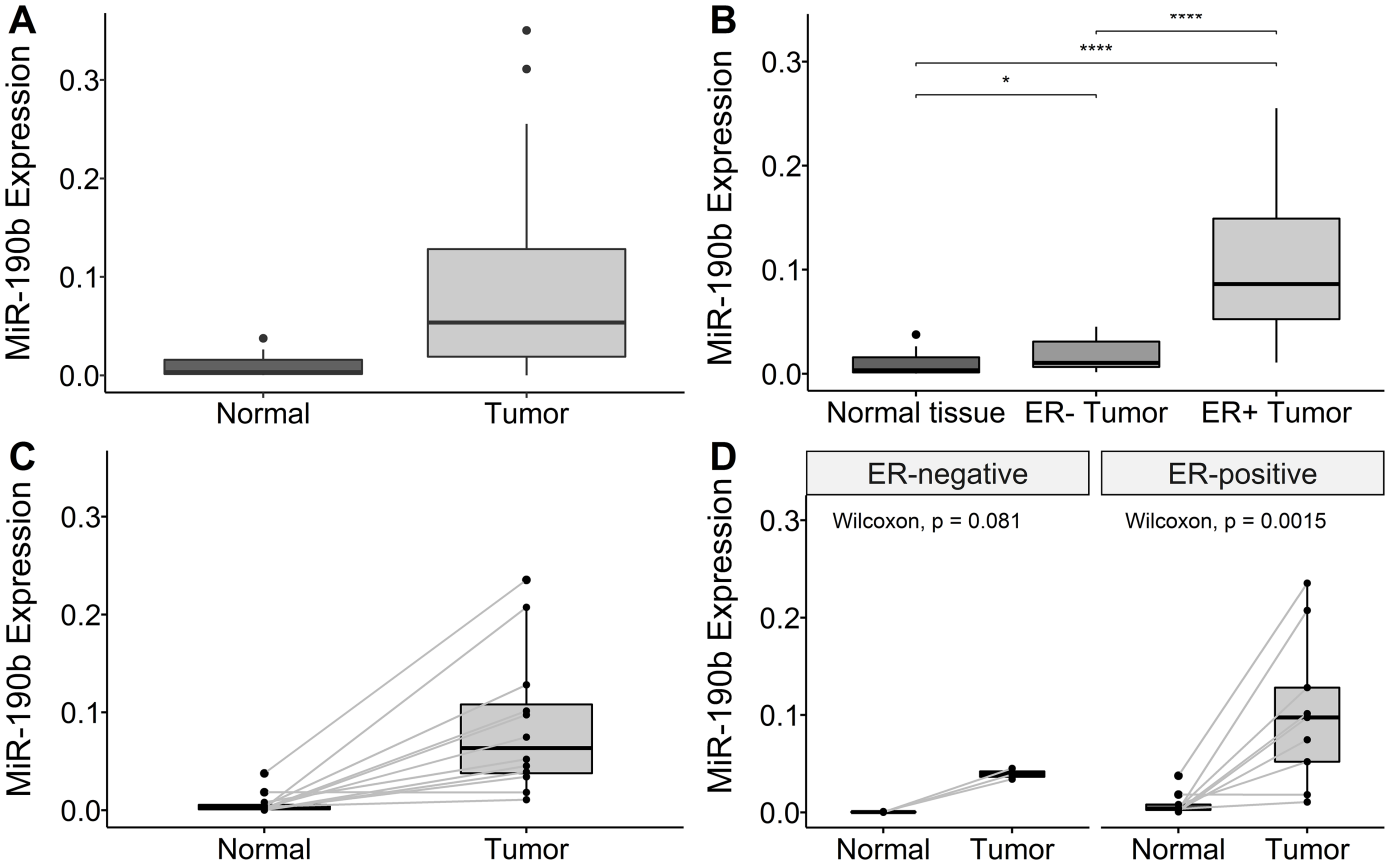
MiR-190b expression in our cohort. (**A**) Overall miR-190b expression is higher in tumor samples (*n* = 62) compared to normal breast tissue (*n* = 15) (Wilcoxon rank sum test *P* = 2.18e–05). (**B**) ER+ breast cancers (*n* = 30) significantly over-express miR-190b comparing to ER– (*n* = 13) and normal tissue (*n* = 15) (Kruskal–Wallis, *P* = 9.13e–07 with Dunn‘s multiple comparison). ^*^ < 0.05, ^**^ < 0.001, ^***^ < 0.0001). (**C**) Overall pairwise miR-190b expression is higher in tumor samples compared to normal breast tissue (*n* = 13) (Wilcoxon signed rank test, *P* = 0.003). (**D**) There is significant over-expression in ER+ tumors compared pairwised (*n* = 9) to normal samples (Wilcoxon signed rank test, *P* = 0.013). No significant difference was found in ER– tumors pairwised compared to normal tissue (*n* = 3) (Wilcoxon signed rank test, *P* = 0.18).

MiR-190b expression was measured in breast tumors (*n* = 62) and normal breast tissue samples adjacent to breast tumor sites (*n* = 15). The breast tumor samples showed overall higher levels of miR-190b expression compared to normal breast tissue (Wilcoxon rank sum test *P* = 2.18e–05, Median values Tumors 0.054, Normal Tissue 0.003) (Figure 2A). Paired samples of normal and tumor tissue were available from 13 individuals of the cohort. Overall pairwised miR-190b expression in tumors was significantly higher compared to normal tissue (Wilcoxon signed rank test, *P* = 0.003, Median values Tumors 0.052, Normal 0.003) (Figure 2C).

While investigating miR-190b expression by ER tumor status, we observed both ER+ and ER– tumors to over-express miR-190b comparing to normal breast tissue. ER+ tumors also significantly over-express miR-190b compared to ER– tumors (Kruskal–Wallis, *P* = 9.13e–07 followed by Dunn‘s multiple comparison, Median values ER+ 0.086, ER– 0.01) (Figure 2B). Differences in miR-190b expression between ER– tumors and normal tissue was not statistically significant in pairwised testing while it was statistically significant for ER+ tumors (Figure 2D). Following the confirmation that ER+ breast tumors over-express miR-190b, we proceeded to investigate whether there was a distinction in over-expression between breast cancer subtypes.

### MiR-190b expression in LumA vs LumB

**Figure 3:**
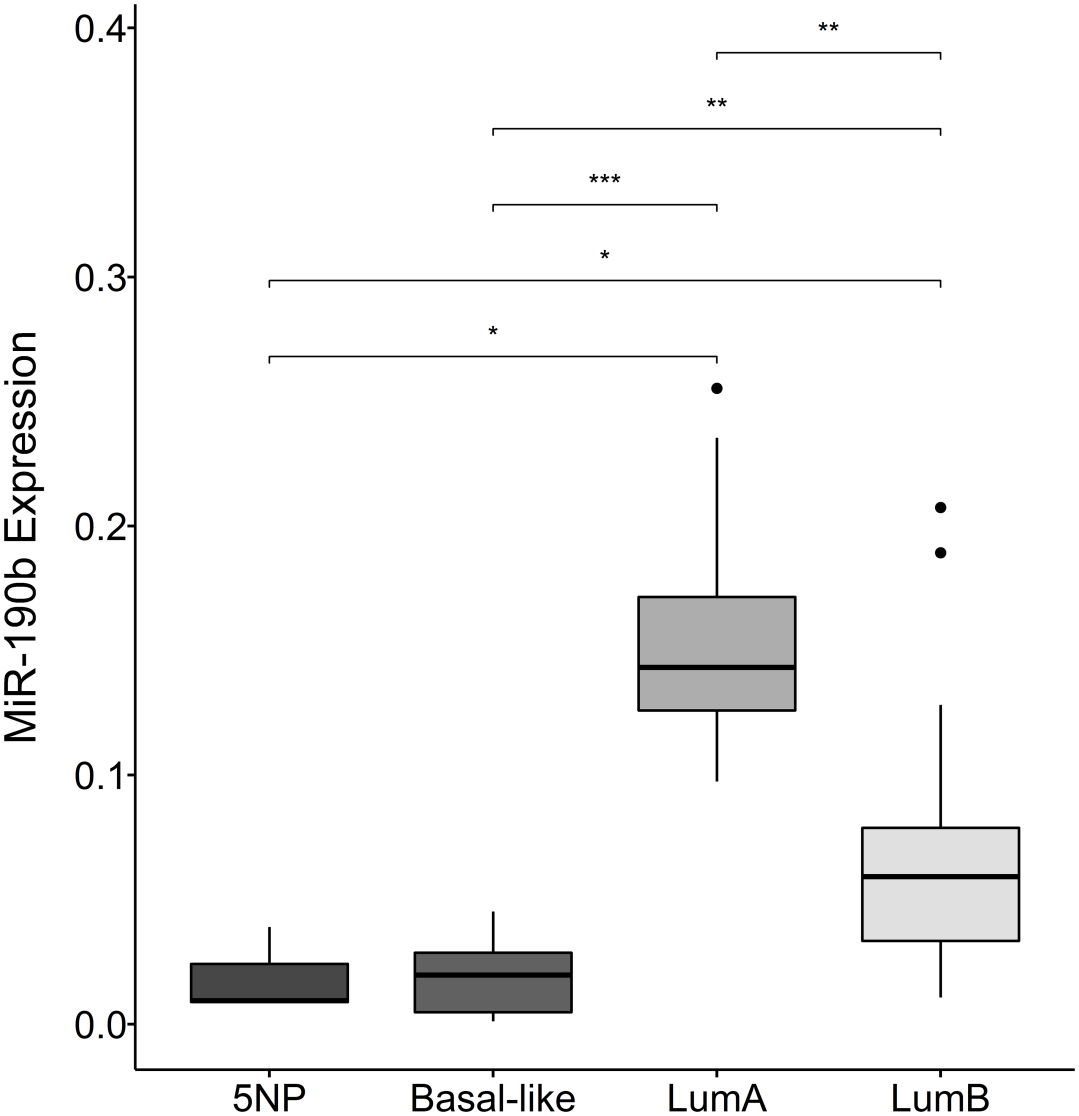
MiR-190b expression in breast cancer subtypes. There is a significant elevation in expression within the subtypes defined as ER+ (lumA (*n* = 8) and lumB (*n* = 18)), compared to ER– subtypes (Basal-like (*n* = 11) and 5NP (*n* = 3)) (Kruskal–Wallis, *P* = 1.0e–04). Furthermore, lumA significantly over-expresses MiR-190b compared to lumB. ^*^ < 0.05, ^**^ < 0.001, ^***^ < 0.0001.

We observed a significant increase in expression within the subtypes defined as ER+, namely LumA and LumB, compared to the ER– subtypes Basal-like and 5NP (Kruskal–Wallis, *P* = 1.0e–04 followed by Dunn‘s multiple comparison, Median values LumA 0.14, LumB 0.059, Basal-like 0.02, 5NP 01.01). We also observed that LumA breast cancers significantly express higher levels of miR-190b comparing to LumB. There was no evident difference in expression within ER– subtypes (Basal-like & 5NP) (Figure 3). Due to unavailability of tumor RNA samples from individuals diagnosed with the HER2 subtypes, a comparison with this particular subtype could not be implemented. Following our observations that there is a distinction in miR-190b expression within ER+ breast cancer subtypes, we investigated whether there is a difference in miR-190b promoter methylation based on ER status and whether there is a further division within breast cancer subtypes.

### MiR-190b methylation status in breast tumors

**Figure 4:**
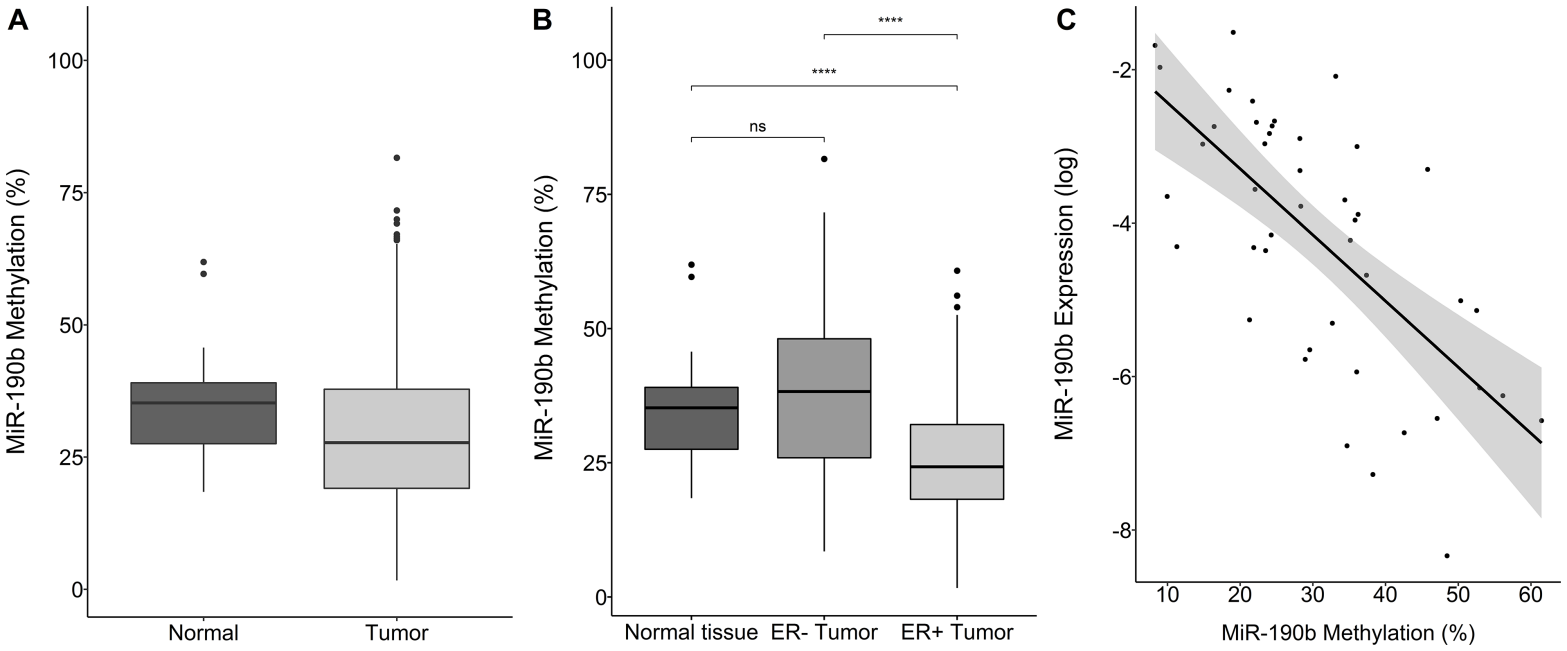
MiR-190b‘s methylation status by sample type, breast cancer subtype and expression. (**A**) Overall tumor miR-190b methylation (*n* = 514) is significantly lower comparing to normal tissue (*n* = 72) (Wilcoxon rank sum test, *P* = 1.18e–04). (**B**) ER+ breast tumors (*n* = 309) have significantly lower miR-190b methylation status compared to ER– tumors (*n* = 113) and normal tissue (*n* = 72) (Wilcoxon signed rank test, *P* = 9.70e–10). (**C**) Expression status of miR-190b is negatively correlated with it‘s methylation status in breast tumors (Spearman’s rho = –0.62, *P* = 1.83e–06) ^***^ < 0.0001.

We pyrosequenced DNA from breast tumors (*n* = 514) and normal tissue samples (*n* = 72). Tumors were significantly less methylated comparing to normal tissue (Wilcoxon rank sum test, *P* = 1.18e–04, Median values Tumors 27.71, Normal tissue 35.24) (Figure 4A). Significant differences were observed between 43 paired tumor and normal tissue samples (Wilcoxon signed rank test, *P* = 0.046, Median values, Tumor 24.04, Normal 36.29) (Supplementary Figure 3A).

From paired RNA and DNA from individual tissue samples (*n* = 63) we observed that miR-190b‘s methylation status is negatively-correlated to its expression in tumors (Spearman’s rho = –0.62, *P* = 1.83e–06) (Figure 4C).

ER+ tumors were significantly less methylated comparing to normal tissue (Wilcoxon signed rank test, *P* = 9.70e–10, Median values ER+ 24.25, Normal Tissue 35.24/ Wilcoxon rank sum, *P* = 0.006, Median values ER+ 30.45, ER– 36.19) (Figure 4B and Supplementary Figure 3B). Methylation in ER– tumors was equivalent to normal tissue (Wilcoxon signed rank test, *P* = 0.067, Median values ER– tumors 38.26, Overall Normal tissue 35.24, Wilcoxon rank sum, *P* = 0.48, Median values ER–36.19, Normal 35.24) (Figure 4B and Supplementary Figure 3B). Following these observations, we looked into miR-190b‘s methylation status according to subtype.

### MiR-190b methylation status in breast cancer subtypes LumA and LumB

**Figure 5:**
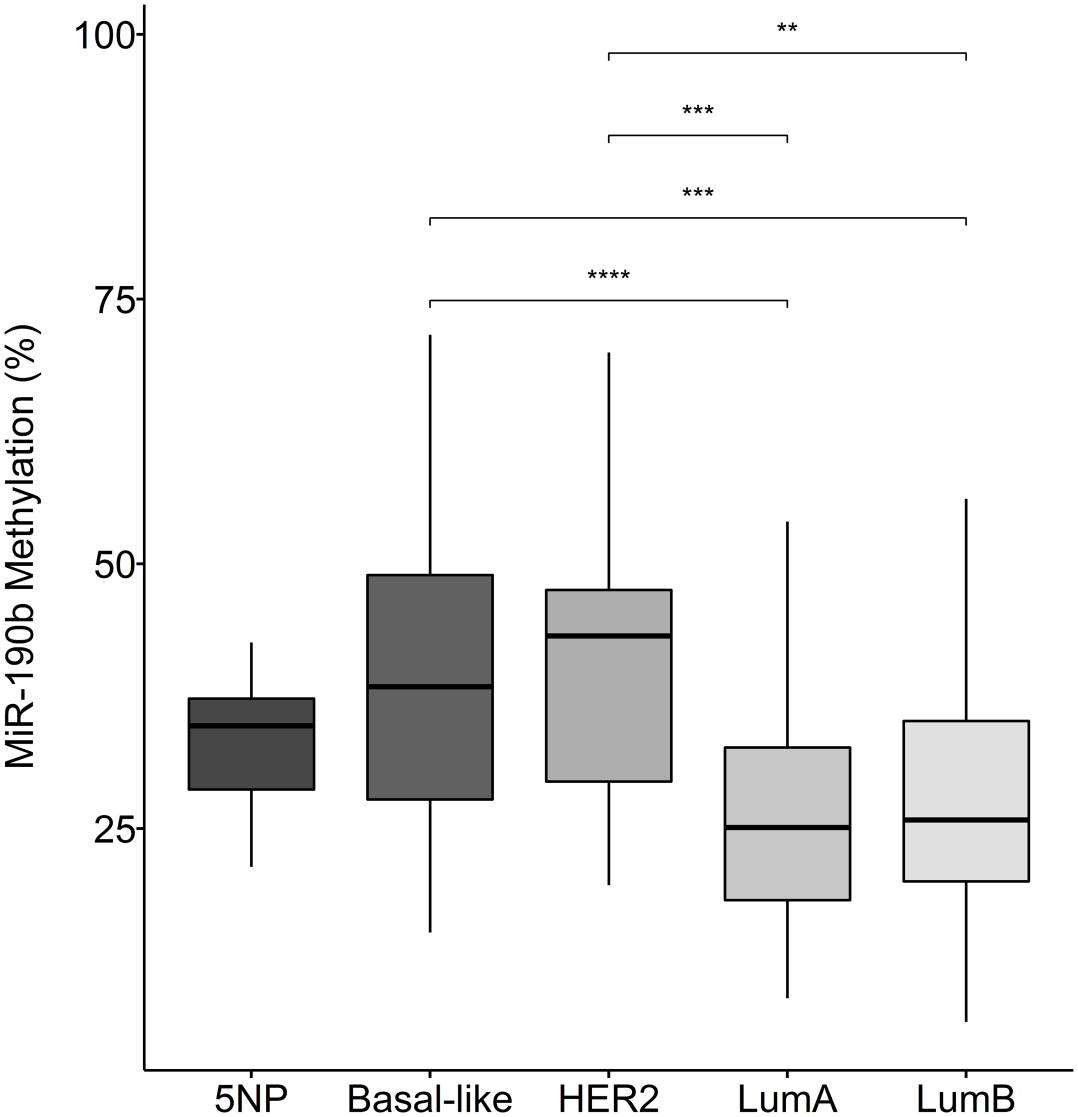
MiR-190b methylation in breast cancer subtypes. The ER+ breast cancer subtypes LumA (*n* = 70) and lumB (*n* = 61) have significantly lower miR-190b methylation comparing to the ER– subtypes HER2 (*n* = 15), Basal-like (*n* = 32) and 5NP (*n* = 7) (Kruskal–Wallis test, *P* = 1.19e–05 followed by Dunn‘s multiple comparison, ^**^ < 0.001, ^***^ < 0.0001).

We observed that the ER+ subtypes LumA and LumB are significantly less methylated comparing to the ER– breast cancer subtypes Basal-like, HER2 and 5NP (Kruskal–Wallis test, *P* = 1.19e–05 followed by Dunn‘s multiple comparison, Median values LumA 25.10, LumB 25.82, Basal-like 28.41, Her2 43.20, 5NP 34.71) (Figure 5).We did not observe any significant differences in methylation status within subtypes of the same ER status, indicating that decreased methylation occurs in both the ER+ subtypes, LumA and LumB, in a similar manner (Figure 5). These findings strongly support our hypothesis that ER+ breast cancers over-express miR-190b via a reduction in promoter methylation. We subsequently sought to understand whether our findings are relevant with respect to clinical parameters.

### MiR-190b promoter methylation in association with clinical parameters

Table 1 summarizes the clinical and pathological characteristics of our cohort. We define hypo-methylation of miR-190b below (or equal to) 20% methylation on the basis of the 1st quartile of the distribution in tumor samples. MiR-190b hypo-methylation was not found to be significantly prevalent with any clinical parameters other than ER status where roughly 87% of miR-190b hypo-methylated tumors were ER+ (Supplementary Table 2).

**Table 1:** Clinical and pathological characteristics of our cohort

		**Overall**
*n*		639
Estrogen receptor status (%)	Neg	117 (26.8) (^*^20.7)
	Pos	319 (73.2) (^*^56.4)
	Unknown	130 (^*^22.9)
Progesteron receptor status (%)	Neg	198 (45.4) (^*^35)
	Pos	238 (54.6) (^*^42)
	Unknown	130 (^*^23)
HER2 status (%)	Neg	159 (61.6) (^*^28.1)
	Pos	99 (38.4) (^*^17.5)
	Unknown	308 (^*^54.4)
Ki67 status (%)	Neg	106 (42.7) (^*^18.7)
	Pos	142 (57.3) (^*^25.1)
	Unknown	318 (^*^56.2)
Nodal Metastases (%)	No	31 (47.7) (^*^5.5)
	Yes	34 (52.3) (^*^6)
	Unknown	501 (^*^88.5)
Year of diagnosis (median [IQR])		1992.00 [1989.00, 1995.00]
Tumor size in mm (median [IQR])		22.00 [15.00, 33.00]
Grade (%)	1	11 (11.6) (^*^1.9)
	2	40 (42.1) (^*^7.1)
	3	41 (43.2) (^*^7.2)
	Other	3 (3.2) (^*^0.5)
	Unknown	471 (^*^83.3)
TNM Stage (%)	I	13 (20.3) (^*^2.3)
	IIa	20 (31.2) (^*^3.5)
	IIb	13 (20.3) (^*^2.3)
	IIIa	9 (14.1) (^*^1.6)
	IIIb	6 (9.4) (^*^1.1)
	IV	3 (4.7) (^*^0.53)
	Unknown	502 (^*^88.7)
Age of diagnosis (median [IQR])		56.00 [46.00, 66.00]
Sampletype (%)	Normal	73 (^*^11.4)
	Tumor	566 (^*^88.6)
Subtype (%)	5NP	7 (3.5) (^*^1.2)
	Basal-like	37 (18.6) (^*^6.5)
	HER2	15 (7.5) (^*^2.7)
	LumA	74 (37.2) (^*^13.1)
	LumB	66 (33.2) (^*^11.7)
	Unknown	367 (^*^64.8)

Percentages are in brackets of known val 1.

### MiR-190b and *BRCA2*^*999del5*^

**Figure 6:**
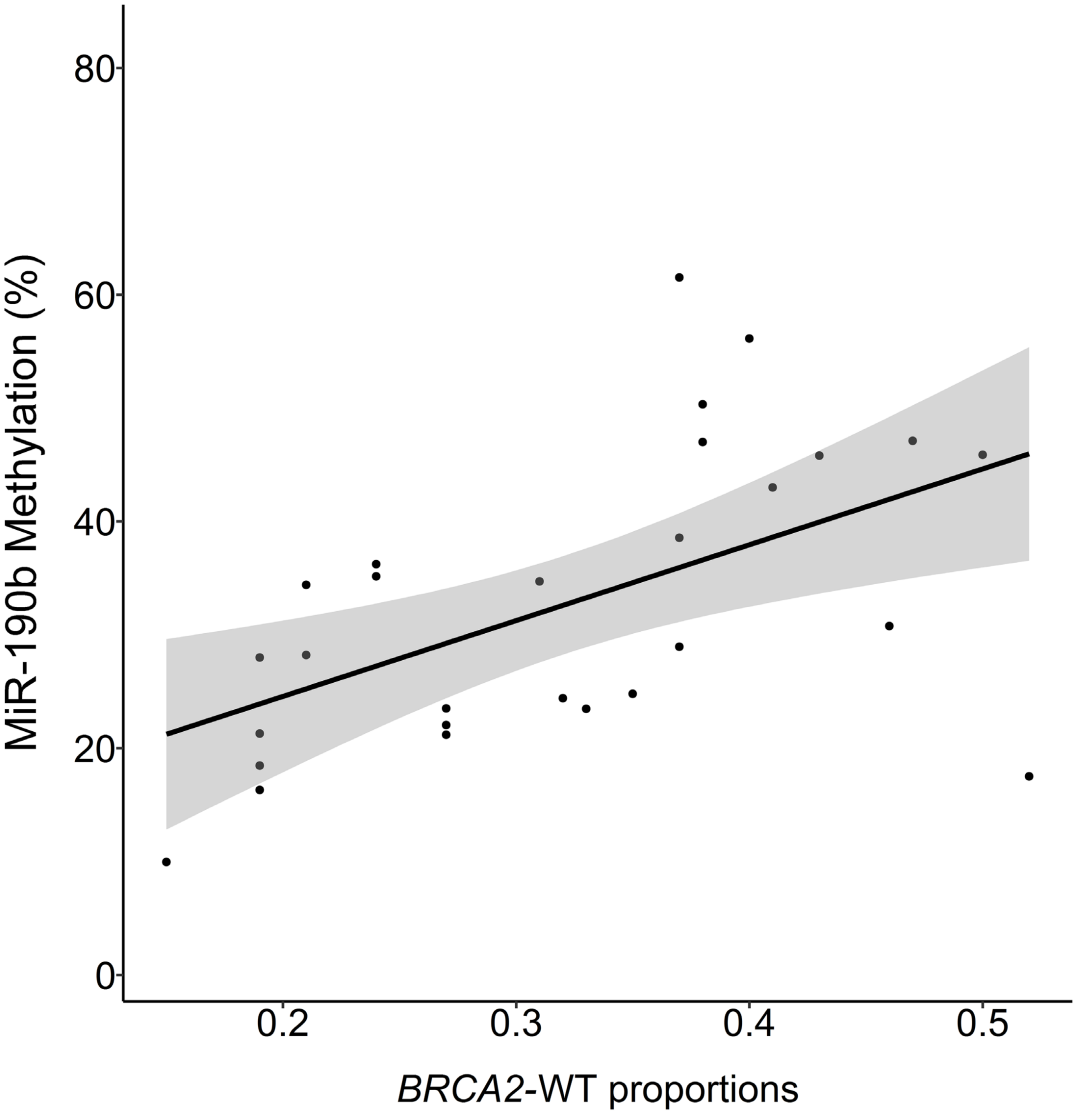
MiR-190b methylation and proportions of BRCA2-wild-type to mutant allele in BRCA^999del5^ carrier tumors. BRCA2 proportion of 0.5 indicates 1:1 proportions of wt-BRCA2 and 999^del5^ alleles, values under 0.5 suggest loss of wt-BRCA2. The proportions are positively correlated with miR-190b methylation (Multivariate linear regression corrected for ER status, age at diagnosis and year of diagnosis, *P* = 0.008, *R*^2^ = 0.45, *n* = 23).

The patients in our cohort had previously been screened for the Icelandic *BRCA2*^*999del5*^ founder mutation and *BRCA2* allele proportions within mutation carriers, enabling us to analyse these factors with regard to miR-190b methylation. When analysing *BRCA2* allele proportions we found correlation between BRCA2 loss of heterozygosity (LOH) and decreased miR-190b methylation in *BRCA2*^*999del5*^ tumors. (Multivariate linear regression corrected for ER status, age at diagnosis and year of diagnosis, *P* = 0.008, *R*^2^ = 0.45, *N* = 28) (Figure 6). Only four tumors derived from carriers were miR-190b hypo-methylated. Looking into miR-190b hypo-methylation with regard to overall *BRCA2*^*999del5*^ mutation status in ER+ patients, we found that miR-190b hypo-methylation events are significantly fewer in ER+ tumors arising in *BRCA2*^*999del5*^ mutation carriers compared with sporadically arising ER+ tumors (Table 2).

**Table 2:** MiR-190b hypomethylation status according to *BRCA2* mutation status in ER+ tumors

	MiR-190b non hypomethylation (%)	MiR-190b hypomethylation (< 20%)
*BRCA2*^*999del5*^ carriers	26 (81.3%)	6 (18.7%)
WT	173 (62.7%)	103 (37.3%)
		*P* = 0.038, *Χ*^2^ = 4.32

**Figure 7:**
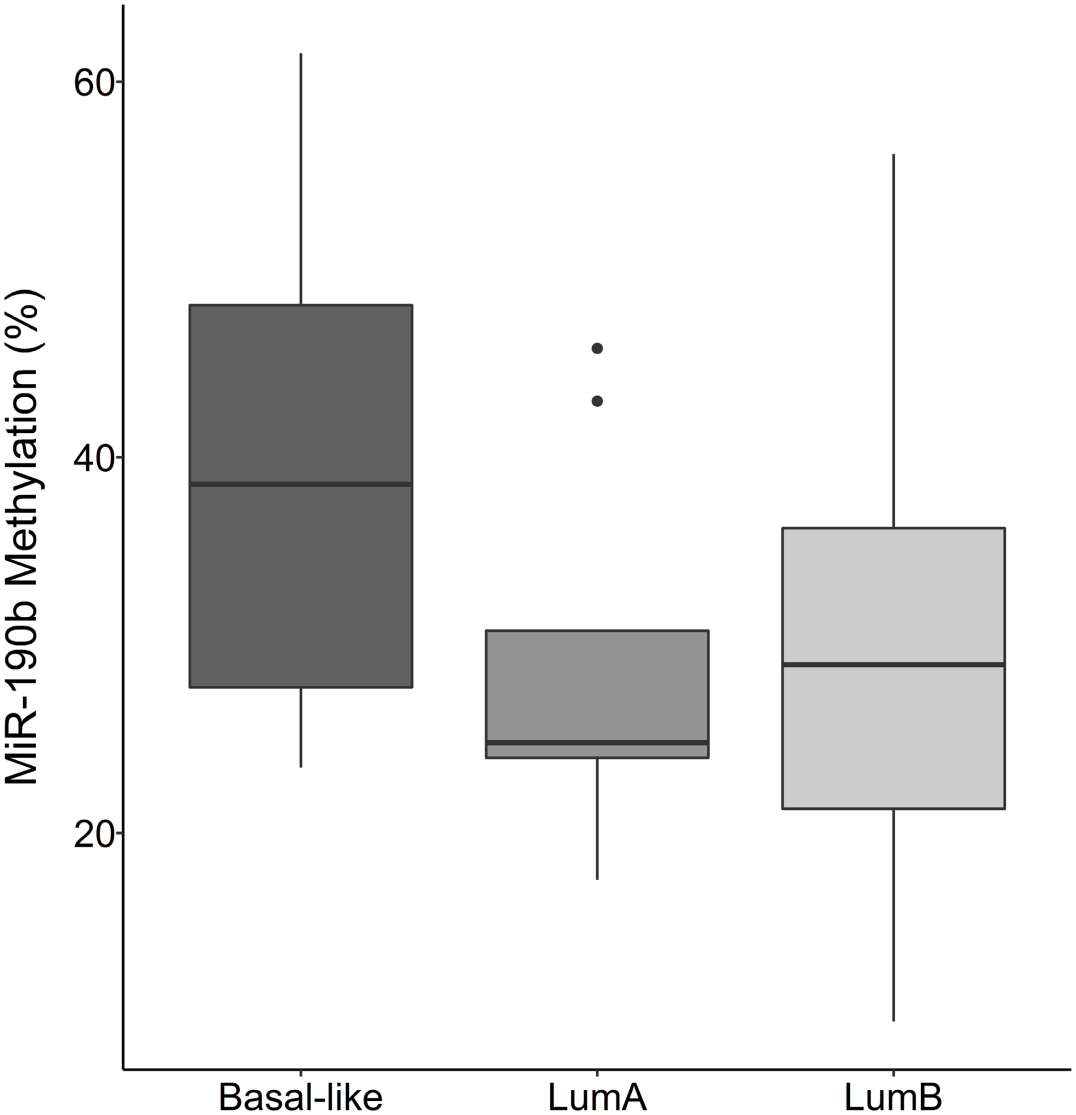
MiR-190b methylation by breast cancer subtypes in BRCA2 999^del5^ carriers. There is no statistically significant difference in miR-190b methylation between subtypes in patients with the BRCA2 999^del5^ founder mutation Basal-like (*n* = 7), LumA (*n* = 9), LumB (*n* = 17) (Kruskal–Wallis test, *P* = 0.383 followed by Dunn‘s multiple comparison).

There was no statistically significant difference in miR-190b methylation between breast cancer subtypes in *BRCA2*^*999del5*^ mutation carriers (Figure 7) (Kruskal–Wallis test, *P* = 0.383 followed by Dunn‘s multiple comparison).

### Breast cancer specific survival and miR-190b

**Figure 8:**
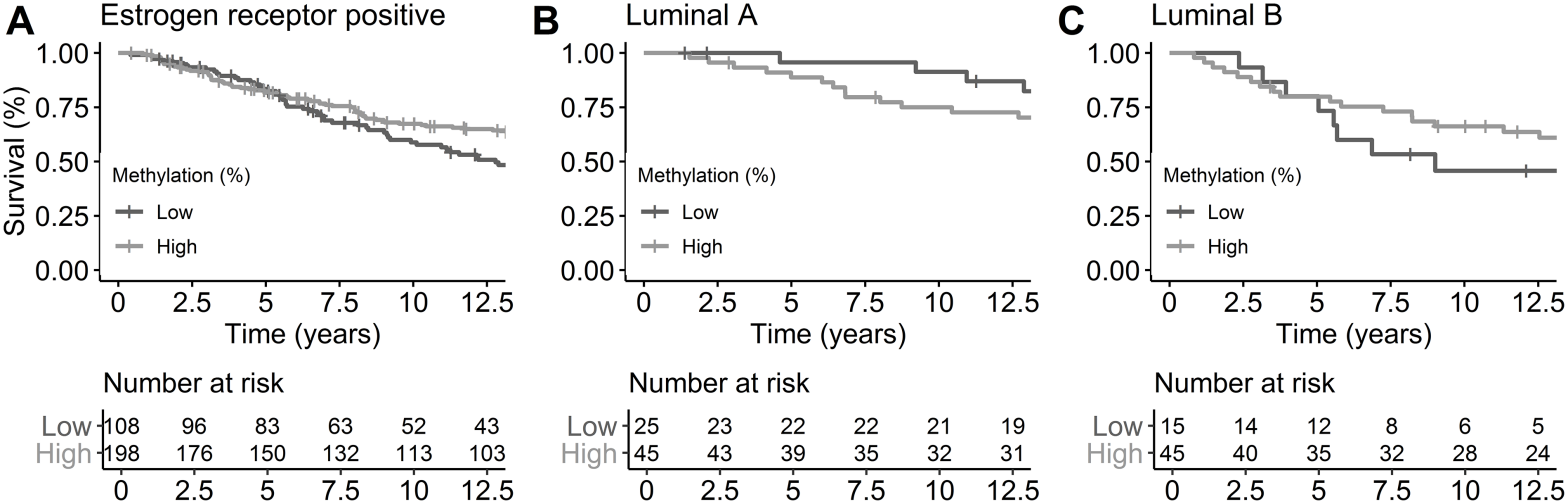
Breast cancer-specific survival by ER status and subtypes by miR-190b methylation status. Cutoff of high and low methylation was set at 20%. (**A**) ER+ breast cancer patients (HR = 1.35, 95% CI 0.95-1.93, *P* = 0.092). (**B**) LumA breast cancer patients (HR = 0.29, 95% CI 0.09-0.91, *P* = 0.034). (**C**) LumB breast cancer patients (HR = 1.71, 95% CI 0.76-3.86, *P* = 0.194). All analysis are corrected for age at diagnosis, year of diagnosis and BRCA2 mutation status.

To determine whether miR-190b methylation status has prognostic value, we carried out survival analysis using a multivariate Cox proportional hazards regression for breast cancer specific survival over time. Maximum follow-up was approximately 43 years with a mean follow-up of 13 years. Breast cancer specific survival times did not differ in ER+ tumors with respect to miR-190b hypo-methylation (HR = 1.35, 95% CI 0.95-1.93, *P* = 0.092) (Figure 8A). After dividing the ER+ cohort into subtypes (LumA and LumB) we observed that LumA patients showed significantly better survival with low methylation (HR = 0.29, 95% CI 0.09-0.91, *P* = 0.034) (Figure 8B). There was no statistically significant difference in LumB (HR = 1.71, 95% CI 0.76-3.86, *P* = 0.194) (Figure 8C) though a trend of poorer breast cancer specific survival in hypo-methylated patients could be seen. Overall survival analysis of miR-190b expression from The Cancer Genome Atlas (TCGA) confirms our findings as overall ER+ and LumB tumors do not show a statistically significant difference in high versus low expression, while LumA does (Supplementary Figure 4).

These results indicate that low miR-190b methylation may be protective for individuals of subtype LumA. There was no significant difference in survival of *BRCA2*^*999del5*^ mutation carriers based on miR-190b methylation status, this is likely due to a small sample size as a trend can be seen (HR = 0.30, 95% CI 0.39-4.69, *P* = 0.469) (Supplementary Figure 5).

We analysed breast cancer specific survival in patients diagnosed with ER– tumors and found overall poorer survival in individuals with low miR-190b methylation (HR = 2.25, 95% CI 1.13-4.46, *P* = 0.020). This result is likely unrelated to miR-190b expression as ER– tumors do not show over-expression of miR-190b according to Figure 2. Owing to lack of statistical power survival analysis for ER– subtypes was not performed (HER2, Basal-like, 5NP).

## DISCUSSION

We show that miR-190b is collectively over-expressed and hypo-methylated in ER+ breast derived tumors and cell lines, indicating that cellular alterations occur in ER+ tumors leading to its upregulation. Interestingly, LumA tumors have significantly higher miR-190b expression compared to LumB while hypo-methylation status remains similar between the two subtypes. There may thus be additional factors facilitating miR-190b expression after loss of methylation within LumA tumors which requires further research. Heterogeneity in miR-190b methylation can be detected in paired normal and tumor samples, as some tumors have an increase in miR-190b methylation. This may be due to different developmental factors in tumor formation, leading to a drive of methyltransferase activation/deactivation within tumors. The biological implications of alterations within the epigenetic machinery can thus be changes in phenotypes that cannot be detected with conventional genomic sequencing. As such, loss of miR-190b methylation leads to occurring overexpression when the genetic code remains unchanged.

MiR-190b methylation is relevant for breast cancer specific survival in patients with LumA cancers. Although miR-190b hypo-methylation was detected in ER– tumors, over-expression did not occur. In spite of these observations, individuals diagnosed with ER– tumors showed worse survival when their tumors exhibited miR-190b hypo-methylation. This likely due to other causes than over-expression of miR-190b. Certain sequence-specific transcription factors needed for inducing high expression levels of miR-190b, possibly involving the estrogen-receptor and/or it‘s cofactors, are likely absent in ER– tumors.

MiR-190b is located within the first intron of transcript 222 of *Tropomyosin 3 (TPM3)* (ENST00000515609) (Supplementary Figure 1), a small transcript with poorly known function [22]*.* Intragenic DNA methylation has been shown to modulate alternative splicing through MeCP2 and promoting exon recognition [23]. Hypo-methylated introns have also been inversly correlated with higher levels of intron retention in mRNA from where it is located [24]. Previous studies on the *TPM3* gene have shown it to be involved in tumorigenesis, migration, and invasion in hematopoietic tumors as well as expression of MMP family members and EMT-like activators in gliomas [25, 26]. Alterations of TPM3 on the protein level due to miR-190b hypo-methylation could thus be leading to a more agressive phenotype in ER– tumors as data from TCGA shows no general correlation between miR-190b and changes in TPM3 expression (Data not shown) [27–29]. TPM3 expression in TCGA remains similar between subtypes (Data not shown). Our speculations are thus that TPM3 expression regulation may be carried out similarly, yet fine-tuned based on subtype, leading to the abovementioned changes being found in one but not the other.

**Figure 9:**
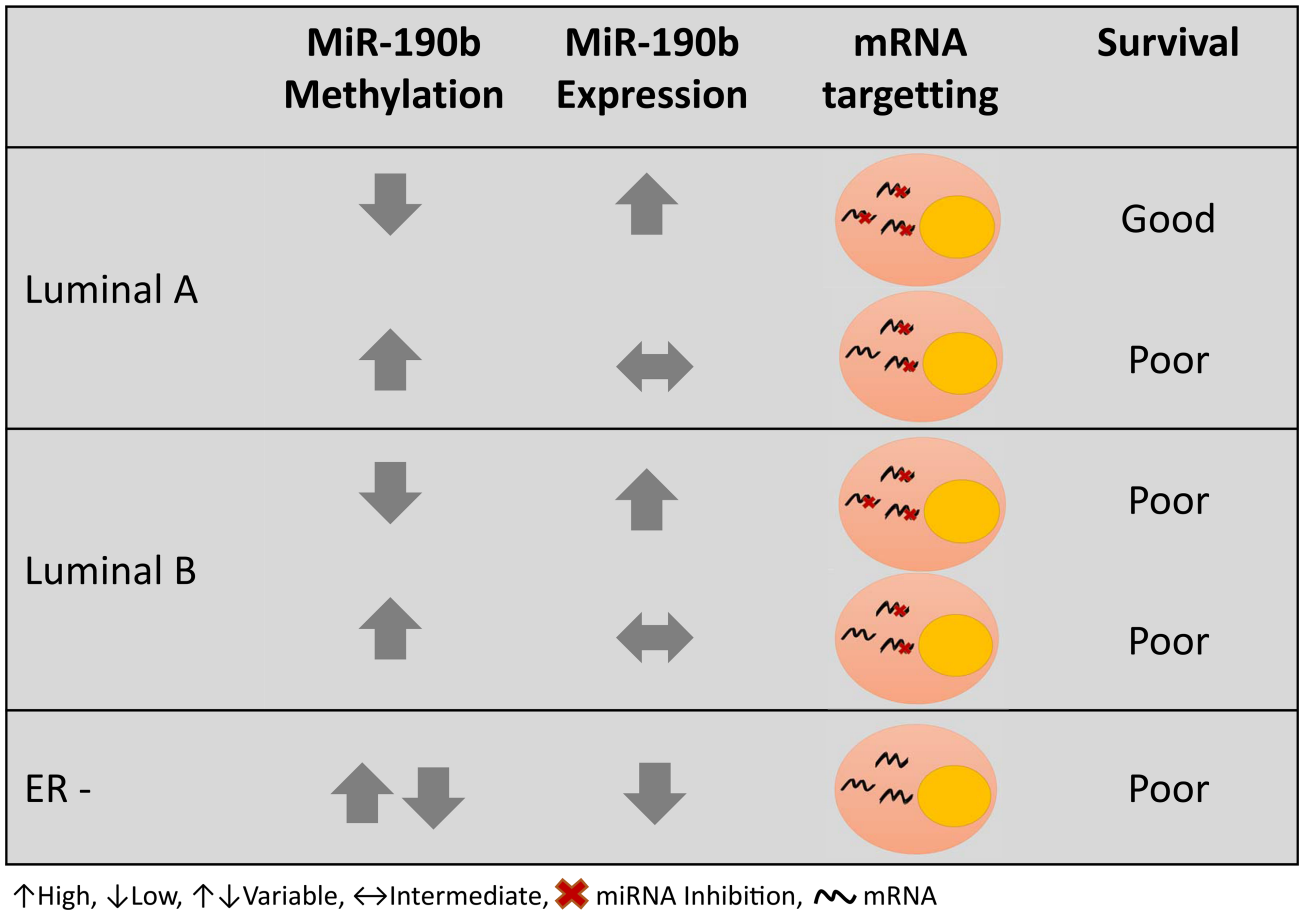
Graphical summary of miR-190b methylation and expression in different breast cancer subtypes and prognosis.

Subtype specific survival analysis performed on LumA and LumB patients suggests that miR-190b is over-expressed in hypo-methylated ER+ breast tumors, though only leading to a more favourable prognosis in LumA patients (Figure 9). This may be due to targetting of oncogenes or oncogenic co-factors. We subsetted our cohort to look into any differences in clinical parameters between hypo-methylated LumA tumors and LumB tumors but did not detect any difference between them. We furthermore looked into clinical parameters comparing hypo- and methylated (>20%) samples within LumA and LumB tumors separately. That, as well, did not lead to any further findings.

MiR-190b hypo-methylation was less frequent in *BRCA2*^*999del5*^ carrier tumors. Results showing *BRCA2* loss of heterozygosity in *BRCA2*^*999del5*^ tumors with decreasing miR-190b methylation lead us to believe that different developmental events due to the mutation may be occuring compared to non-mutated tumors. Data from TCGA showed no correlation between miR-190b and *BRCA2* expression on either the mRNA or protein level of BRCA2 (Data not shown). With regard to survival, we did not see a statistical difference in breast cancer specific survival in the patients with *BRCA2*^*999del5*^ mutation due to lack of power. It is worth noting that some results, specifically when looking into the *BRCA2*^*999del5*^, are based on few values. Nonetheless, a trend of worse survival was seen in individuals with low miR-190b methylation as was observed in patients with ER– and LumB tumors (data not shown). Unsurprisingly, 24 of the 34 tumors of known subtypes in *BRCA2*^*999del5*^ carriers were LumB and Basal-like. Loss of BRCA2 has been linked to increased sensitivity to DNA damaging chemotherapeutic agents, due to loss of homologus recombination DNA repair [30]. Previous assumptions were that most tumors from *BRCA2* germline mutation carriers had locus-specific LOH [31]. Recent studies have however shown otherwise, demonstrating that up to roughly 50% of tumors associated with *BRCA2* germline mutations lack locus-specific LOH [32]. Investigating miR-190b with regard to *BRCA2* LOH in mutation carriers may thus be biologically relevant when researching this phenotype. Common miRNA target predictions show that direct miR-190b targetting of *BRCA2* is unlikely, and further research is needed to evaluate the abovementioned associations.

Roughly 70% of all breast cancers are diagnosed as ER+, which can also be seen in our patient group. 35% of our ER+ samples are miR-190b hypo-methylated indicating high prevalence of this trait. These events may be suggestive of early breast cancer development towards ER positivity. Early diagnosis is an important factor for improved prognosis, and as previously mentioned, ER+ tumors are most commonly treated using agents inhibiting the estrogen receptor or hormone level [33]. MiR-190b is thus an interesting potential tool for investigating developmental aspects regarding ER+ tumors. Additional research of miR-190b hypo-methylated and miR-190b methylated tumors of the same subtype are key to understanding potential targettable factors within these subgroups. Transcriptional differences between LumA and LumB tumors are particularly intriguing and may lead to further characterization of ER+ subtypes.

## MATERIALS AND METHODS

### Patient groups

The group we used in this study was derived from a sample collection previously screened for the Icelandic founder *BRCA2*^*999del5*^ germ line mutation [34]. DNA samples were available from 549 primary invasive breast tumors, of which 62 were derived from *BRCA2*^*999del5*^ carriers. 96 tumor RNA samples were available, of which 67 were paired with available DNA. 26 of RNA samples came from *BRCA2*^*999del5*^ carriers, of those, 23 were paired with DNA. 13 RNA samples of normal breast tissue, pairing with tumors, were available. 71 DNA samples of normal breast tissue were available, of which 13 samples were paired with tumors. All tumor samples were examined by a pathologist at the Department of Pathology Landspitali-University Hospital, Iceland. DNA was isolated from freshly frozen tumors by phenol-chloroform/proteinase K extraction or, when freshly frozen tumors were not available, from formalin-fixed and paraffin embedded tumors by xylene-deparaffinization and lysis/proteinase K digestion. Normal breast tissues were acquired from a distal location of the cancer tissue, deriving from the same individuals in our cohort at the time of surgery (*n* = 71). A total of 16 breast derived cell lines were used in the study (Supplementary Table 1).

Patient data was provided by the Icelandic Cancer Registry [35] as of January 2018, in collaboration with pathologists at the Department of Pathology Landspitali-University Hospital, Iceland. Clinical staging was according to the TNM system (tumor size and nodal status), while histological grade was assessed by the Nottingham system. The study was carried out in compliance with permission from the Icelandic Data Protection Commission (2006050307) and Bioethics Committee (VSNb2006050001/03-16).

### Cell culture

The cell lines used in this study were obtained from the American Type Culture Collection (ATCC). The cells were cultured in DMEM (CAMA-1, MDAMB-468, MCF-7, MCF-10A, MDAMB-231 and SKBr-3), RPMI (HCC-38, Bt-474, HCC1937, HCC1428, HCC1500, T-47D, ZR-75-1) or Leibovitz’s L-15 (MDA-MB-435, MDA-MB-436, MDA-MB-134-VI) with added 10% serum (+penicillin/streptomycin) and other supplements according to ATCC guidelines.

### DNA methylation analysis by pyrosequencing

PyroMark Q24 pyrosequencing instrument was used to analyse information on DNA methylation for the candidate promoter region of miR-190b. The first CpG, 166 bases, upstream from miR-190b’s stem-loop sequence was analysed (Supplementary Figure 1). We made use of Qiagen’s Pyromark Assay Design 2.0 to design primer sequences for the analysis.

Bisulfite conversion of the DNA samples was carried out using the EZ DNA methylation-gold kit (Zymo Research). Pre-PCR amplification of our sequence of interest was carried out by using Hot-Start polymerase (Immolase, Bioline) in a Veriti (Applied Biosystems) thermocycler. The target was amplified using forward primer 5′-GGAGAGTTATTTTTTTGAGGAAGGGTATTG-′3 and reverse primer 5′-(Btn)ACCCTACCAAATATTCTTCCTAATTTA-′3 with a PCR reaction mixture consisting of: 14,5 µl DNase/RNase-free water, 0,5 µl each of forward and reverse primer (10 µM), 0,8 µl dNTP (100 µM), 1,1 µl MgCl2, 2 µl 10xbuffer, 0.1 µl Immolase DNA Polymerase, 0,5 µl bisulfite converted DNA. The reaction cycle consisted of denaturation at 95°C for 10 min, 50 cycles of amplification at 95°C for 30 sec, 56°C for 30 sec and 72°C for 30 sec, and cycle completion with extension at 72°C for 10 min. The target PCR product was then sequenced by synthesis using sequencing PyroMark Q24 reagents and sequencing primer 5′-TTTTAAGATAGTTAGTTTTTGTTTA-′3.

The signal data derived from PyroMark Q24 pyrosequencing of CpG sites, the incorporation of T and C, are analysed by Qiagen´s PyroMark Q24 software using an in-built CpG methylation analysis feature. The output reflects the degree of CpG methylation in percent values, from 0 to 100% methylation.

### TaqMan miR-190b quantitative PCR in breast cancer samples and breast derived cell lines

RNA samples were isolated from freshly frozen tumors using Trizol reagent (ThermoFisher). Additionally, RNA samples derived from simultaneous RNA/DNA isolation by the AllPrep DNA/RNA/miRNA universal Kit (Qiagen) method were also included in the cohort. Total RNA concentration was quantified by using NanoDrop™ One/One^C^ Microvolume UV-Vis Spectrophotometer (ThermoFisher). In total, 77 (62 tumors and 15 normal breast tissues) RNA samples were available for expression analysis of which 62 samples had corresponding DNA for methylation analysis.

MiRNA expression levels were measured by quantitative RT-qPCR using FAM labelled pre-designed and pre-optimized TaqMan Advanced miRNA Assay (Applied Biosystems, cat: A25576). Using TaqMan Advanced miRNA cDNA synthesis kit (cat: A28007), the RNA samples from patients were reverse transcribed in a 10 ng concentration in a total final volume of 30 µl. Each step of cDNA synthesis was carried out as described in the manufacture protocol. Subsequently, 5 µl of the resulting cDNA was pre-amplified in a final volume of 50 µl as detailed in the protocol, following the described cycling mode. Prior to performing the RT-PCR reactions, efficiency analysis was implemented in a 3x fold cDNA dilution series of 8 dilutions, starting from undiluted sample, to set a cycle range for which the samples should not exceed and guarantee reaction efficiency. RT-PCR reaction mix ratios were prepared according to protocol to a final volume of 5 µl. Each reaction contained: 2,5 µl 2x Fast Advanced Master Mix, 0,25 µl TaqMan Advanced miRNA Assay (20x), 1 µl RNase-free water, 1,25 µl cDNA in a dilution range within efficiency curve limits. BioRad CFX384 Touch™ Real-Time PCR Detection System was used, in 384 well plate format, the reaction cycle was as follows: denaturation at 95°C for 20 sec, 40 cycle amplification at 95°C for 1 sec and 59°C for 20 sec. Amplification curves were linear for all samples and ranged within 90–110% efficiency. Each sample was repeated three times in triplicate with repetitions of samples exceeding standard deviation 0,5 per run. To normalize expression levels, miR-190b expression was measured relative to miR-425 and miR-423. Sample dilution was fixed between test and control genes. Negative control was added to each reaction run. Relative gene expression was calculated using the formula 2^(-1^*^(Average test gene expression- Average control gene expression)). When combining reference genes for miR-190b we used the geometric mean between expression outcomes of the controls. Threshold cycle levels were fixed between each run within the exponential phase of the amplification curves. The Cq upper limit was set to 36 where Cq values equal to or greater than that was considered as not expressed.

### TMA (tissue microarray) expression analysis

Tissue microarrays (TMAs) were previously constructed and analysed for ER (1D5, DAKO), PR (PgR 636, DAKO), HER-2 (HercepTest Kit, DAKO) and Ki67 (MIB1, DAKO) by immunohisochemisty (IHC) [36, 37]. ER and PR negativity were defined where staining was seen in less than 1% of the tumor cells. HER-2 was defined as positive only where intense membranous staining was seen, following guidelines provided by the antibody manufacturer.

### *BRCA2* allele-specific quantitative PCR

TaqMan *BRCA2* allele-specific quantitative PCR was previously performed and analysed [12]. Proportions of *BRCA2* wild type (wt) relative to 999del5 *BRCA2* mutated alleles were quantitatively measured by TaqMan qPCR (7500 Realtime OCR system, Applied Biosystems). A single *BRCA2*-specific minor groove-binding probe (MGB-probe) 5′-end labelled with FAM and a nonfluorescent quencher (NFQ) at the 3′-end, a single *BRCA2*-specific forward primer, and two allele-specific reverse primers were used: Forward primer: 5′-CATGATGAAAGTCGTAAGAAA-3′, Reverse primer (mut): 5′-CATGACTTGCAGCTTCTCTTTGTG-3′, Reverse primer (wt): 5′-CATGACTTGCAGCTTCTCTTTGAT-3′, TaqMan-MGB probe: 5′-TTTATCGCTTCTGTGACA-3′.

### Statistics

To compare methylation status between groups we used Wilcoxon’s rank sum test for independent samples, Wilcoxon’s signed rank test for paired samples, and non-parametric Kruskal–Wallis with post-hoc analysis using Dunn‘s multiple groups comparison [38]. Benjamini Hochberg method for false discovery rate was used for multiple comparisons correction. To adjust for confounding factors between clinical variables that resulted significant we used multivariate linear regression for modelling the relationship between methylation status and given clinical features. We divided miR-190b methylation outcomes into quartiles resulting in 4 groups of methylation status ranging from low to high. We performed chi-square test for independence between methylation status and clinical features. Spearman’s non-parametric correlation analysis was performed to determine the association between methylation status and gene expression. Kaplan–Meier method was applied to generate survival curves. Relative hazards were estimated in multivariate analyses using the Cox proportional hazards model, adjusting for potential confounding factors such as ER status, year of diagnosis and age at diagnosis [39, 40]. Breast cancer-specific survival is defined as time from diagnosis to end of follow-up or death. Survival analyses were performed using Survival package in R. Patients who died of other causes than breast cancer were censored at date of death. The cut off for defining high vs low methylation status was set at 20% methylation. Cut off was determined at the lower quartile of tumor methylation status (19.29%) and rounded up to 20%. Follow-up was until January 1st, 2018. Statistical analysis was carried out using R program [41] and packages [42–44]. Generation of Supplementary Figure 1 was carried out using Bioconductor packages Gviz, Genomic Ranges and biomaRt [45–47].

## CONCLUSIONS

We have demonstrated that miR-190b hypo-methylation events occur in ER+ positive breast cancers and are associated with increased breast cancer specific survival in LumA patients. MiR-190b‘s association with favorable survival in LumA patients suggests that miR-190b has an active role in these tumors, indicating that there might be transcriptional differences within the ER+ subtypes that are yet to be identified as clinically relevant. The high prevalence of miR-190 hypo-methylation in ER+ breast tumors indicates early onset occurences of miR-190b activation, leading us to assume miR-190b may have a role in fine-tuning developmental pathways in tumorigenesis. We have shown that reduced miR-190b methylation correlates with locus specific LOH in *BRCA2*^*999del5*^ mutation carriers. Less frequent hypo-methylation in carriers indicates developmental drive away from miR-190b hypo-methylation and thus restricting over-expression. Further research on miR-190b is needed to identify its target genes. Such identification may be a useful tool in recognizing relevant biological and developmental pathways in breast cancer.

## SUPPLEMENTARY MATERIALS



## Abbreviations

ATCC: American Type Culture Collection
CI: Confidence interval
ER: Estrogen receptor
LOH: Loss of heterozygosity
Lum A: Luminal A
Lum B: Luminal B
miRNA: microRNA
mRNA: messengerRNA
OR: Odds ratio
PR: Progesterone receptor
TCGA: The cancer genome Atlas
TMA: Tissue microarray
TNM: Tumor size and nodal status
TPM3: Tropomyosin 3
UTR: untranslated region
WT: Wild type
5-NP: Quintuple negative
