# Supplementary material for: CpG promoter hypo-methylation and up-regulation of microRNA-190b in hormone receptor-positive breast cancer
Source: Oncotarget. 2019 Jul 23;10(45):4664–78. doi: 10.18632/oncotarget.27083 (PMC6659800; doi:10.18632/oncotarget.27083)
Supplement: Supplementary file 1 [file oncotarget-10-4664-s001.pdf]

CpG promoter hypo-methylation and up-regulation of microRNA-190b in hormone receptor-positive breast cancer

SUPPLEMENTARY MATERIALS

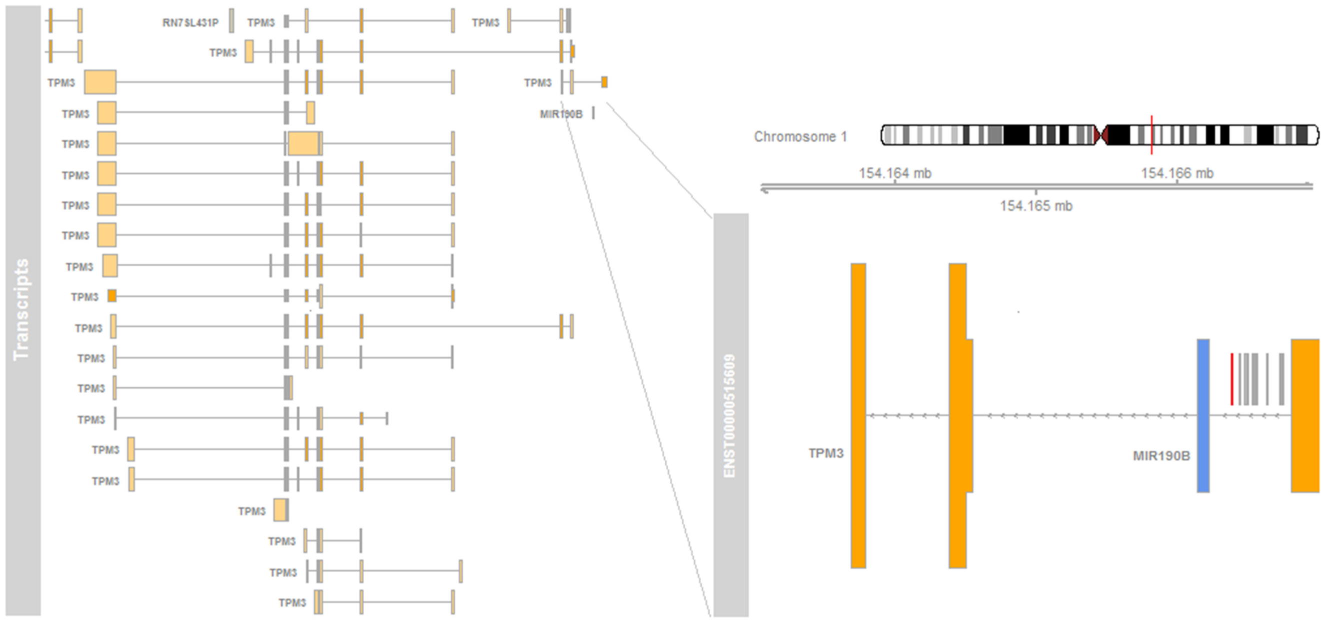

Supplementary Figure 1: MiR-190b's genetic location and upstream CpG's. MiR-190b is situated within the TPM3 transcript ENST00000515609. CpG's upstream from MiR-190b within the transcript are labelled as gray ticks. The CpG analysed in this study is labelled as a red tick.

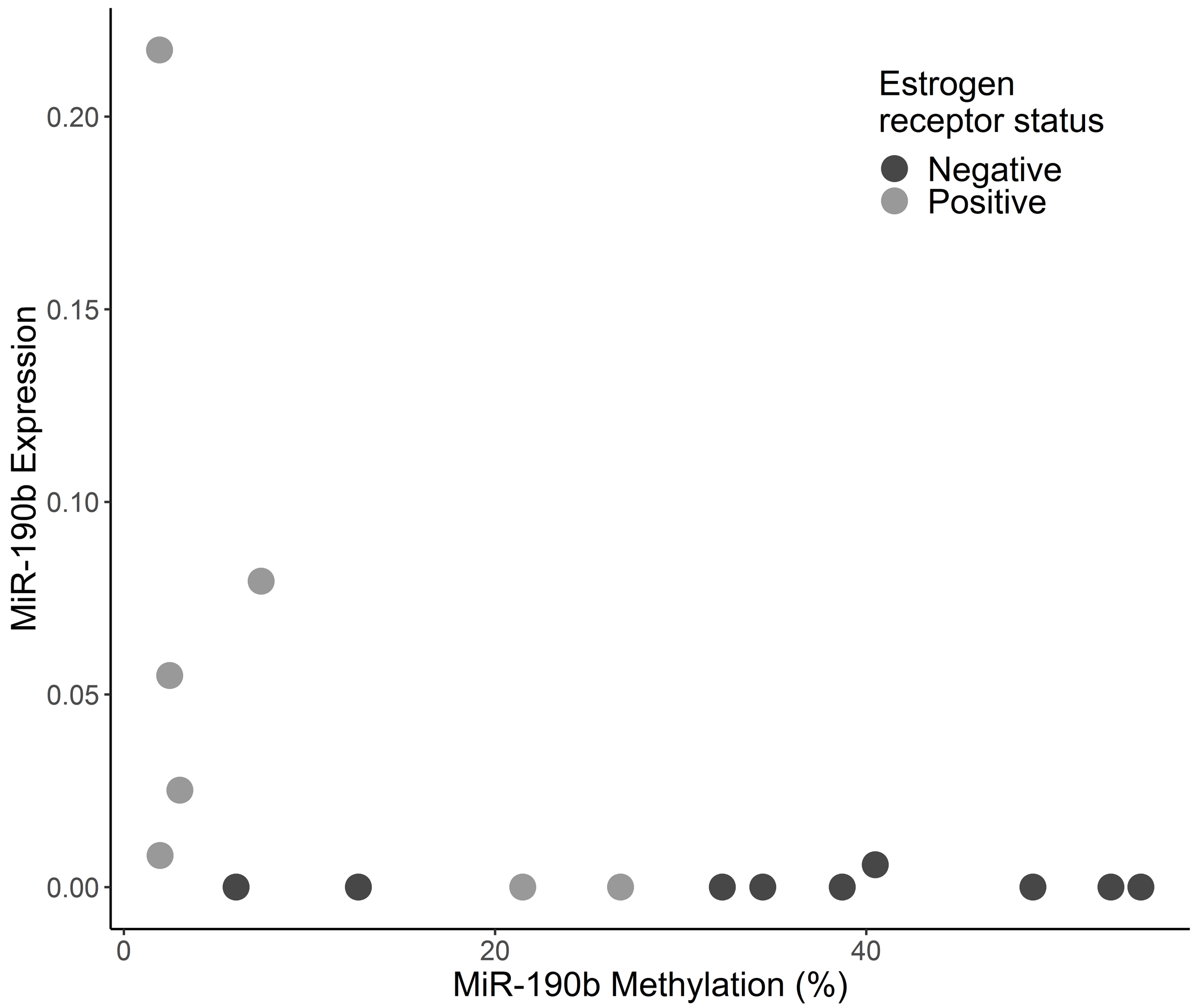

Supplementary Figure 2: MiR-190b mRNA expression and CpG promoter methylation in breast cancer cell lines. Spearman's rho correlation analysis was significant, $R^2 = -0.68$, $p = 0.004$, $n = 16$.

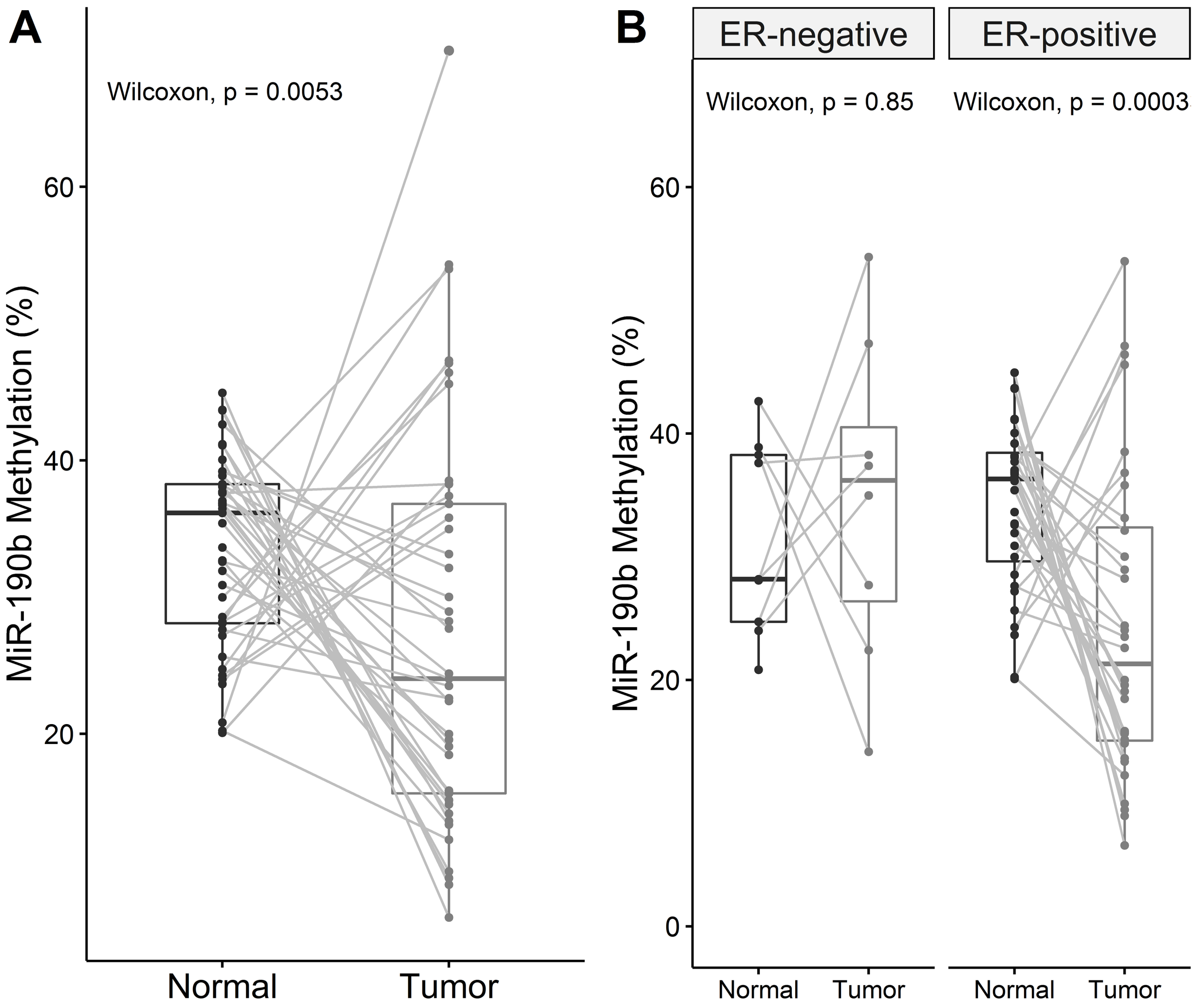

Supplementary Figure 3: MiR-190b pairwise methylation status in tumor and normal tissue. (**A**) pairwised comparison of miR-190b methylation status between breast tumors and normal breast tissue ($n = 43$) (Wilcoxon signed rank test, $P = 0.046$). (**B**) After dividing tumor tissue into their according ER status. ER+ tumors ($n = 32$) have significantly lower miR-190b methylation comparing to normal breast tissue (Wilcoxon signed rank test, $P = 0.006$). MiR-190b expression in ER- breast tumors ($n = 9$) is not significantly different from normal breast tissue (Wilcoxon rank sum test, $P = 0.48$).

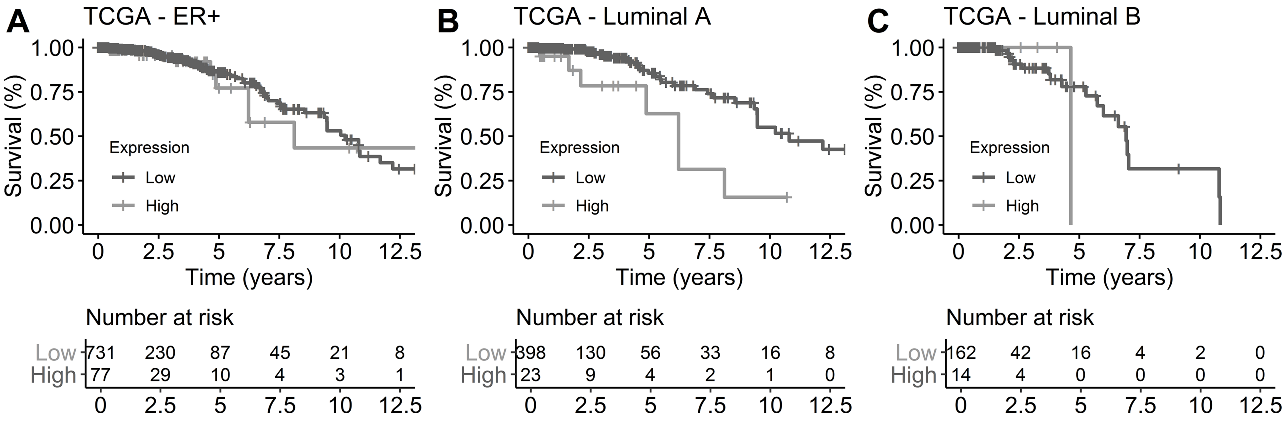

Supplementary Figure 4: Overall survival from TCGA. Cutoff of high and low expression was set at the upper quartile of expression levels for normal tissue from TCGA and analyzed using Cox regression. (**A**) ER+ breast cancer patient (HR = 0.36,CI 0.29-1.34, $P = 0.22$). (**B**) LumA breast cancer patients (HR = 0.26,CI 0.11-0.60, $P = 0.0016$). (**C**) LumB breast cancer patients (HR = 0.63,CI 0.08-5.02, $P = 0.66$). All analysis are corrected for age at diagnosis.

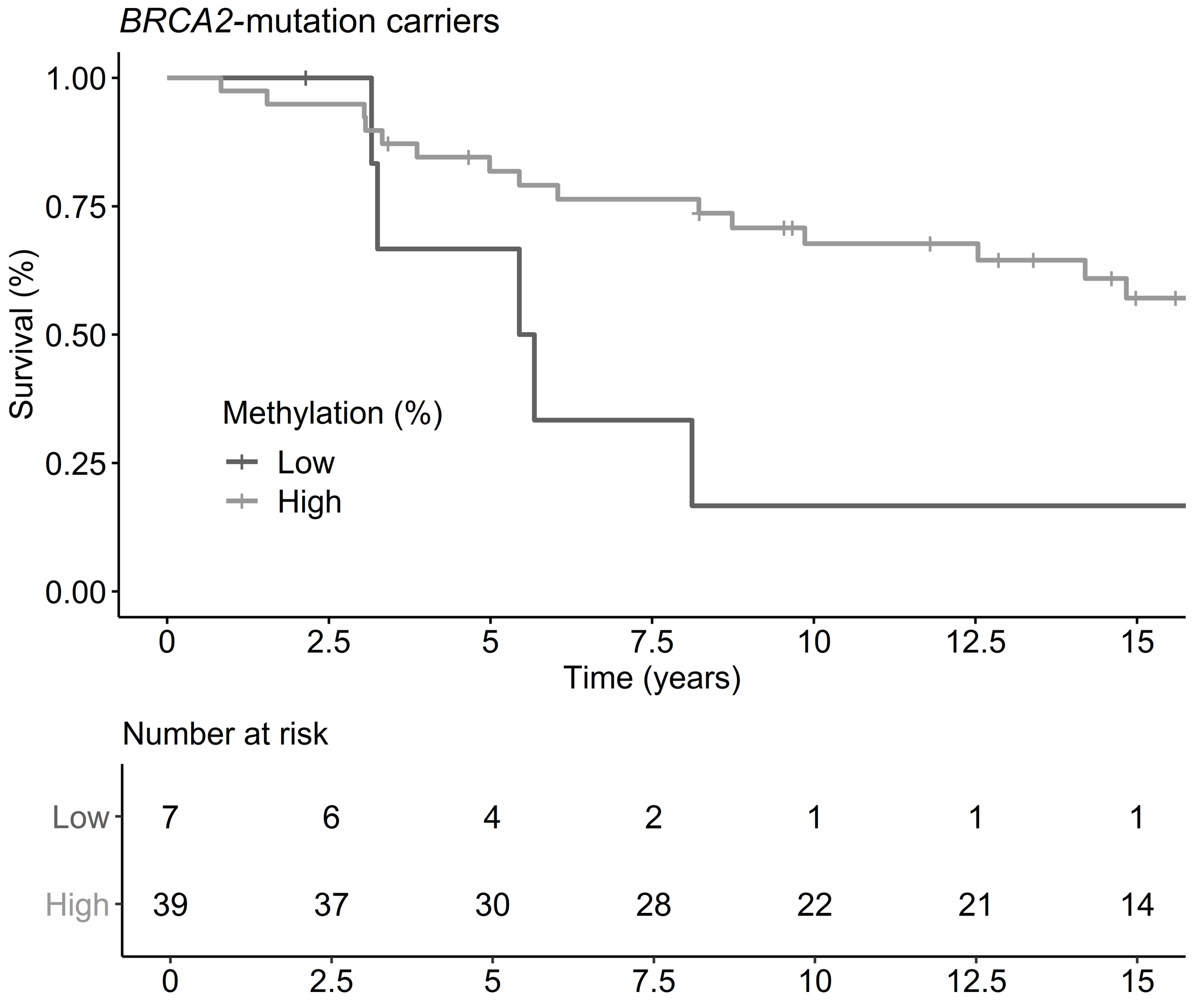

Supplementary Figure 5: Breast cancer specific survival within BRCA2^{999del5} mutation carriers by miR-190b methylation status. (HR = 0.30, 95% CI 0.39-4.69, *P* = 0.469).

Supplementary Table 1: Cell lines

Cell line	Subtype	ER	PR	HER	CK5/6	EGFR	Ki-67
BT-474	LumB(12), Lum(13),	–(12), +(13),	+(12),+(13),	3+(12), +(13)	–(12)	1+(12)	70(12)
CAMA-1	Lum(13)	+(13)	–(13)	–(13)			
HCC38	Basal-like B(13)	–(13)	–(13)	–(13)			
HCC1937	Basal-like A(13)	–(13)	–(13)	–(13)			
HCC1428	Lum(13)	+(13)	+(13)	–(13)			
HCC1500	Basal-like B(13)	–(13)	–(13)	–(13)			
MCF7	LumA(12), Lum(13)	+(12), +(13)	+(12), +(13)	0–1+(12)	–(12)	1+(12)	90(12)
MCF10a	Basal-like(12), Basal-like B(13)	–(12), –(13)	–(12), –(13)	0–1+(12), –(13)	+(12)	2+(12)	30(12)
MDA-MB-134-VI	Lum(13)	+(13)	–(13)	–(13)			
MDA-MB-231	Basal(12), Basal-like B(13)	–(12), –(13)	–(12), –(13)	0–1(12), –(13)	–(12)	1(12)	100(12)
MDA-MB-435	HER2(12), Basal-like B(13)	–(12), –(13)	–(12), –(13)	3+(12), –(13)	–(12)	0(12)	80(12)
MDA-MB-436	Basal-like B(13)	–(13)	–(13)	–(13)			
MDA-MB-468	Basal-like A(13)	–(13)	–(13)	–(13)			
SK-BR-3	HER2(12), Lum(13)	–(12), –(13)	–(12), –(13)	3+(12), +(13)	–(12)	2+(12)	20(12)
T47D	Lum(13)	+(13)	+(13)	–(13)			
ZR-75-1	Lum(13)	+(13)	–(13)	–(13)			

Supplementary Table 2: Tumor characteristics according to miR-190b methylation status

Stratified by miR-190b methylation					
		**Hypo-methylation**	**Methylation**	***p*-value**	**Corrected for ER**
n		156	464		
Estrogen receptor status (%)	Neg	16 (12.8)	97 (32.7)	< 0.001	
	Pos	109 (87.2)	200 (67.3)		
Progesteron receptor status (%)	Neg	42 (33.9)	151 (50.7)	0.002	Insignificant
	Pos	82 (66.1)	147 (49.3)		
HER2 status (%)	Neg	41 (67.2)	106 (58.2)	0.276	
	Pos	20 (32.8)	76 (41.8)		
Ki67 status (%)	Neg	34 (59.6)	66 (37.3)	0.005	
	Pos	23 (40.4)	111 (62.7)		
Nodal Metastases (%)	No	8 (50.0)	20 (45.5)	0.984	
	Yes	8 (50.0)	24 (54.5)		
Year of diagnosis (%)	1969–1989	49 (31.4)	150 (32.3)	0.837	
	1990–1992	27 (17.3)	89 (19.2)		
	1993–1994	39 (25.0)	119 (25.6)		
	1995–2007	41 (26.3)	106 (22.8)		
Tumor size mm (%)	5–15	9 (30.0)	27 (27.8)	0.097	Insignificant
	16–22	10 (33.3)	21 (21.6)		
	23–33	2 (6.7)	27 (27.8)		
	34–Over	9 (30.0)	22 (22.7)		
Grade (%)	1	3 (23.1)	7 (9.1)	0.330	
	2	4 (30.8)	35 (45.5)		
	3	5 (38.5)	33 (42.9)		
	*Other	1 (7.7)	2 (2.6)		
TNM Stage (%)	I	3 (18.8)	9 (18.6)	0.592	
	IIa	4 (25.0)	13 (30.2)		
	IIb	5 (31.2)	8 (18.6)		
	IIIa	3 (18.8)	5 (11.6)		
	IIIb	0 (0.0)	6 (14.0)		
	*IV	1 (6.2)	2 (4.7)		
Age of diagnosis (%)	30–40	10 (6.4)	56 (12.3)	0.013	Insignificant
	41–50	33 (21.2)	126 (27.7)		
	51–60	38 (24.4)	117 (25.7)		
	61–70	39 (25.0)	92 (20.2)		
	70-Over	36 (23.1)	64 (14.1)		
Sample type (%)	Normal	1 (0.6)	70 (15.1)	< 0.001	
	Tumor	155 (99.4)	394 (84.9)		
Subtype (%)	*5NP	0 (0.0)	7 (5.0)	0.008	
	Basal-like	3 (6.8)	29 (20.6)		
	HER2	1 (2.3)	14 (9.9)		
	LumA	25 (56.8)	45 (31.9)		
	LumB	15 (34.1)	46 (32.6)		

*Expected values over 5 was un-obtainable by simplification of groups. Exclusion of these groups does not change outcome.
